# Intervertebral disc degeneration, age, and sex affect the range of motion of the cervical spine

**DOI:** 10.1038/s41598-025-07182-4

**Published:** 2025-07-02

**Authors:** Christian Liebsch, Ann-Kathrin Greiner-Perth, Morten Vogt, Verena Vieres, René Jonas, Annette Kienle, Hans-Joachim Wilke

**Affiliations:** 1 Institute of Orthopaedic Research and Biomechanics, Trauma Research Centre Ulm, Ulm University Medical Centre, Helmholtzstraße 14, 89081 Ulm, Germany; 2 SpineServ GmbH & Co. KG, Ulm, Germany

**Keywords:** Cervical spine, Intervertebral disc degeneration, Age, Sex, Range of motion, In vitro study, Biomedical engineering, Experimental models of disease

## Abstract

Influences of intervertebral disc degeneration, age, sex, and segmental level on the range of motion (ROM) of the cervical spine are widely unknown. This in vitro study aimed to evaluate these effects using large sample sizes. 19 fresh frozen human cervical spines (occiput-T1) were loaded with pure moments of 2.5 Nm. Segmental ROMs were measured by optical motion tracking from C2–C3 to C6–C7. Disc degeneration of these levels was assessed using a validated classification system for the cervical spine. ROMs gradually and significantly decreased with increasing disc degeneration (*p* < 0.05) and showed high negative correlation with age in flexion/extension (rho =  − 0.637) and axial rotation (rho =  − 0.628). In flexion/extension, ROMs were significantly higher in specimens from female donors compared to male (*p* < 0.05). The level did overall not significantly affect ROMs (*p* > 0.05), except for C2–C3, which showed significantly lower ROMs compared to C3–C4 (*p* < 0.05). Increasing disc degeneration and age as well as male sex reduce the ROM of the cervical spine. The relatively continuous decrease of ROM with increasing disc degeneration in all motion directions largely differs from previous findings of our group on the lumbar spine (gradual ROM increase in axial rotation) and the thoracic spine (strong ROM decrease already for mild disc degeneration).

**Supplementary Information:**

The online version contains supplementary material available at 10.1038/s41598-025-07182-4.

## Introduction

Intervertebral disc degeneration is part of the natural ageing process and affects the structural and biomechanical properties of the spine^1,2^. Previous research on the effects of degenerative disc changes has primarily focused on the lumbar spine to investigate potential connections with chronic low back pain^1^. Thus, effects of disc degeneration on the flexibility of the cervical spine have only been little addressed in the literature so far^1–3^, although it is known that disc degeneration is also highly prevalent in the cervical spine^4,5^ and cervical disc degeneration is assumed to contribute to chronic neck pain^6,7^. Moreover, cervical spinal flexibility has been shown to largely differ from the thoracic and lumbar spine^8^, necessitating separate consideration of the specific biomechanical properties of the cervical spine with regard to the effects of degenerative changes of the intervertebral discs. While the normal, undifferentiated segmental range of motion of the cervical spine is largely known from in vivo^9–11^ and in vitro investigations^8,12,13^, the effects of potential influencing factors on the cervical range of motion, such as disc degeneration, age, and sex, are not yet fully understood. However, these factors have to be considered to provide adequate interpretability of data from experimental and clinical studies as well as to ensure high validity of in vitro and in silico models of the healthy and the degenerated cervical spine.

Previous in vivo clinical trials generally reported that the cervical range of motion decreases with increasing disc degeneration^14–16^ and age^15–19^, while effects of sex showed conflicting results with an overall tendency towards reduced cervical range of motion in male subjects compared to females^15–19^. However, experimental in vivo studies on the cervical spine are usually associated with inconsistencies in motion patterns of the participants and substantial intra- and interday variability of range of motion measurement data^20,21^. Moreover, previous in vivo studies either used lateral radiography to investigate flexion/extension movements^15,16^ or external devices to determine the overall cervical range of motion^17–19^, which are usually subjected to higher measuring inaccuracies compared to in vitro measurement techniques. In order to provide reproducible range of motion data with adequate accuracy, in vitro experiments with standardized testing conditions and consideration of the three primary motion planes and all segmental levels are required.

The aim of this in vitro study therefore was to investigate the effect of intervertebral disc degeneration, age, sex, and segmental level on the range of motion of the cervical spine using a high number of motion segments.

## Methods

### Specimens

19 fresh frozen cervical spine specimens (C0-T1) from human donors were acquired from the Institute of Anatomy and Cell Biology of the University of Ulm following approval for their use for in vitro experiments by the ethics committee of the University of Ulm (vote no. 199/99). Donors had given informed consent for use of their body tissue for medical research. Data on donor age and sex was known for all specimens. The mean donor age was 68 ± 15 years, ranging from 44 to 90 years. Eleven specimens originated from female and eight specimens from male donors. The specimens were stored frozen at − 20 °C, thawed at 5 °C prior to preparation and testing, and prepared to solely consist of bony, cartilaginous, and ligamentous tissue. The occiput and the first thoracic vertebra of each specimen were half embedded in polymethylmethacrylate (PMMA, Technovit 3040, Heraeus Kulzer, Wehrheim, Germany). To enhance the fixation of the embeddings, screws were driven into the occipital (C0) as well as through the first thoracic vertebral (T1) and last cervical vertebral (C7) bone. Preparations and experiments described here were carried out in accordance with any relevant guidelines and regulations.

### Intervertebral disc degeneration grading

**Fig. 1:**
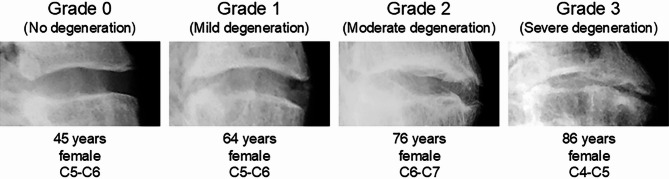
Representative examples of the four grades of cervical intervertebral disc degeneration according to the classification of Kettler et al.^22^ determined in the present in vitro study.

Prior to flexibility testing, lateral radiographs of the entire specimens (C0-T1) were performed in a closed X-ray device (Faxitron 43805N, Hewlett Packard, Palo Alto, CA, USA) using a radiolucent fixture. The specimens were scanned with a tube voltage of 46.5 kV, an exposure time of 60 s, and a source-to-film distance of 60 cm. Based on these radiographs, grading of intervertebral disc degeneration was performed for the segmental levels from C2–C3 to C6–C7 according to the validated and reliability-tested classification system for the cervical spine developed by Kettler et al.^22^. Using this grading scheme, which is based on the evaluation of disc height loss, osteophyte formation, and diffuse sclerosis of the adjacent vertebral bodies, the intervertebral discs were assigned to four degeneration grades (0 = no disc degeneration, 1 = mild disc degeneration, 2 = moderate disc degeneration, 3 = severe disc degeneration, Fig. 1).

### Flexibility testing

To determine the segmental ranges of motion, the specimens were quasi-statically loaded in a well-established spine tester^23^. The testing conditions were according to general recommendations for spinal in vitro testing^24^ and comprised 3.5 cycles of pure moment loading up to 2.5 Nm with a constant loading rate between 1 and 2.5°/s in flexion/extension, lateral bending, and axial rotation. The specimens were used for overall three projects with different research questions. Thus, twelve specimens were tested in flexion/extension and thirteen in lateral bending and axial rotation, respectively. The specimens were prepared and tested at room temperature for an accumulated duration of maximum 20 h and were kept moist using 0.9% saline solution to avoid soft tissue dehydration. Simultaneously with loading, optical motion tracking of each vertebral level from C2 to C7 was performed (Vicon MX13, Vicon Motion Systems Ltd., Oxford, UK; PONTOS, GOM mbH, Braunschweig, Germany) with a measuring accuracy of < 0.1°. The range of motion (ROM) was determined from the moment–angle curve of the third full loading cycle for each motion segment from C2-C3 to C6-C7 and each motion plane using a custom-written MATLAB script (MATLAB R2024a, MathWorks Inc., Natick, MA, USA).

### Statistics

Statistical evaluations of group differences and correlations were performed using SPSS (SPSS Statistics 29.0, IBM Corp., Armonk, USA). Any range of motion data beyond two-fold standard deviation were treated as outliers and excluded from the analysis. For differences between range of motion values of the single degeneration grades and segmental levels as well as for the determination of interdependencies between degeneration grades, age, and segmental levels, the Kruskal–Wallis test with Dunn-Bonferroni post-hoc correction and the two-sided Mann–Whitney U test were performed. The two-sided Mann–Whitney U test was also used to determine differences between range of motion values of age groups (< / > 60 years) and sexes as well as for the evaluation of differences between both sexes regarding degeneration grades and age. Linear relationships between range of motion values and disc degeneration grades as well as age were explored using Spearman correlation coefficients. Correlation coefficients 0.1 <|r|≤ 0.3 were considered as low linear correlation, 0.3 <|r|≤ 0.5 as medium linear correlation, and |r|> 0.5 as high linear correlation according to the recommendation of Cohen^25^. The significance level was set to 0.05 for group comparisons and to 0.001 for correlation analyses.

## Results

### Study collective

**Fig. 2:**
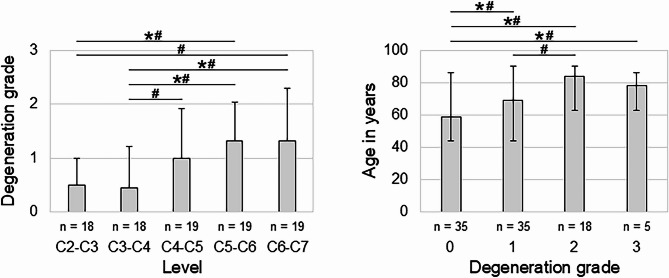
Bar diagrams illustrating the effects of the segmental level on the mean degeneration grade (with standard deviation, left) and the relationship between donor age and degeneration grades (medians with minimum and maximum values, right) for the motion segments tested in this in vitro study. *p < 0.05 (Pairwise Kruskal–Wallis test with Dunn-Bonferroni post-hoc correction), #*p* < 0.05 (Two-sided Mann–Whitney U test).

Of the n = 95 evaluated motion segments, n = 2 were excluded from the analysis due to the above stated outlier criterion. Of the remaining n = 93 motion segments, n = 35 showed no disc degeneration (grade 0), n = 35 mild disc degeneration (grade 1), n = 18 moderate disc degeneration (grade 2), and n = 5 severe disc degeneration (grade 3). The degeneration grades were found to be significantly affected by the segmental level (*p* < 0.05), exhibiting higher degeneration for lower segmental level (Fig. 2 left). Moreover, donor age was significantly increased for higher degeneration grades (*p* < 0.05, Fig. 2 right). No significant differences were found between female and male donors regarding both the degeneration grade and the donor age (*p* > 0.05) (Supplementary figures).

### Effects of intervertebral disc degeneration

**Fig. 3:**
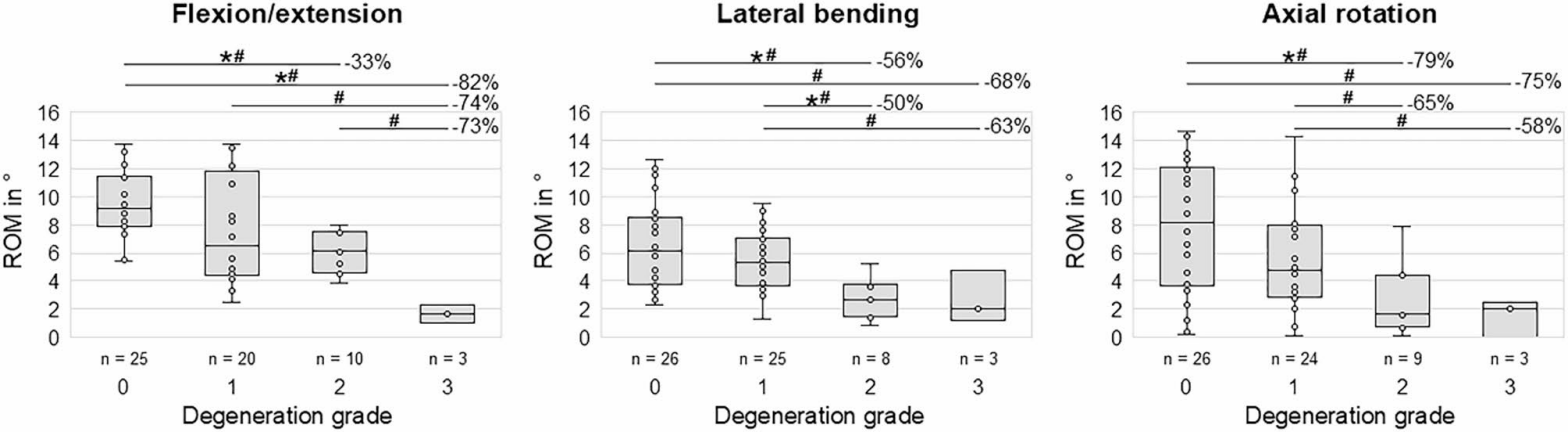
Boxplot diagrams illustrating the effects of intervertebral disc degeneration grades on the segmental range of motion (ROM) of the cervical spine in the three primary motion planes. *p < 0.05 (Pairwise Kruskal–Wallis test with Dunn-Bonferroni post-hoc correction), #*p* < 0.05 (Two-sided Mann–Whitney U test).

Degenerative changes of the intervertebral disc caused a significant decrease of the range of motion in all three motion planes (*p* < 0.05), especially from grade 0 (no degeneration) to grade 2 (moderate degeneration) and grade 3 (severe degeneration), as well as from grade 1 (mild degeneration) to grade 3 (Fig. 3). In flexion/extension, the most pronounced significant decrease of the range of motion was found from grade 2 to grade 3 by 73% (*p* < 0.05), while the highest significant decreases of the range of motion in lateral bending and axial rotation were detected from grade 1 to grade 2, with reductions by 50% and 65% (*p* < 0.05), respectively. From grade 0 to grade 3, the median range of motion was reduced by 82% in flexion/extension, by 68% in lateral bending, and by 75% in axial rotation.

**Fig. 4:**
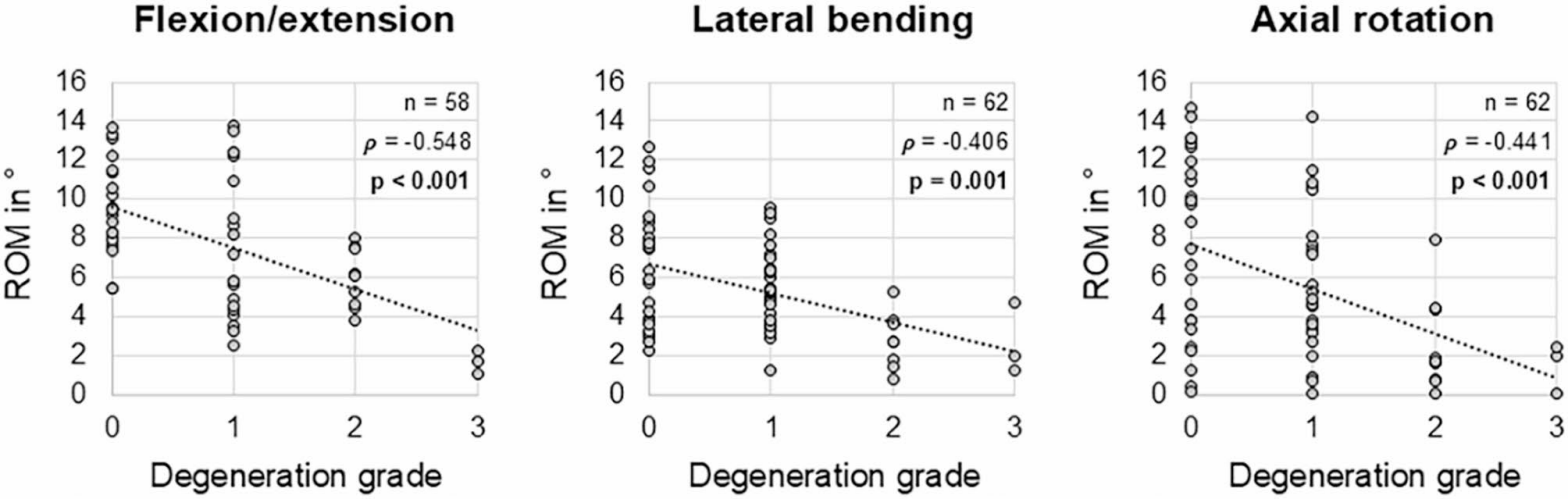
Scatterplot diagrams illustrating the relationship between intervertebral disc degeneration and segmental range of motion (ROM) of the cervical spine in the three primary motion planes. Spearman correlation coefficients (*ρ*) and significance values (*p*) are shown in the upper right corner of each diagram.

The range of motion also showed negative correlation with the degeneration grade in all three motion planes (Fig. 4), meaning that the range of motion decreased with increasing disc degeneration. The correlations were significant for flexion/extension and axial rotation (*p* < 0.001) and tended to be significant in lateral bending (*p* = 0.001). The Spearman correlation coefficients indicated high correlation between the range of motion and the degeneration grade in flexion/extension (*ρ* =  − 0.548) as well as medium correlation in lateral bending (*ρ* =  − 0.406) and axial rotation (*ρ* =  − 0.441). For every next higher grade of disc degeneration, the segmental range of motion decreased by 2.1° in flexion/extension, by 1.5° in lateral bending, and by 2.3° in axial rotation.

### Effects of age

**Fig. 5:**
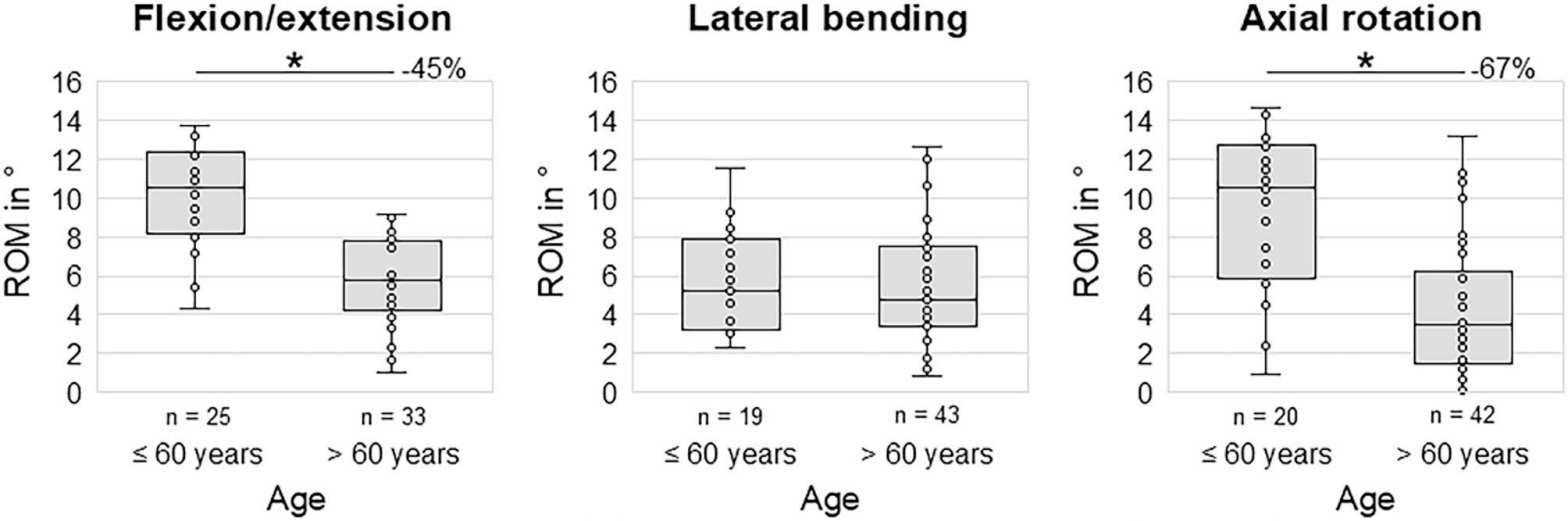
Boxplot diagrams illustrating the effects of age on the segmental range of motion (ROM) of the cervical spine in the three primary motion planes. *p < 0.05 (Two-sided Mann–Whitney U test).

Donor age over 60 years was associated with a significant decrease of the range of motion in flexion/extension and axial rotation compared to the age group under 60 years (*p* < 0.05), leading to a median loss of flexibility of about 45% in flexion/extension and of about 67% in axial rotation (Fig. 5). In lateral bending, no significant age-dependent decrease of the range of motion was observed (*p* > 0.05).

**Fig. 6:**
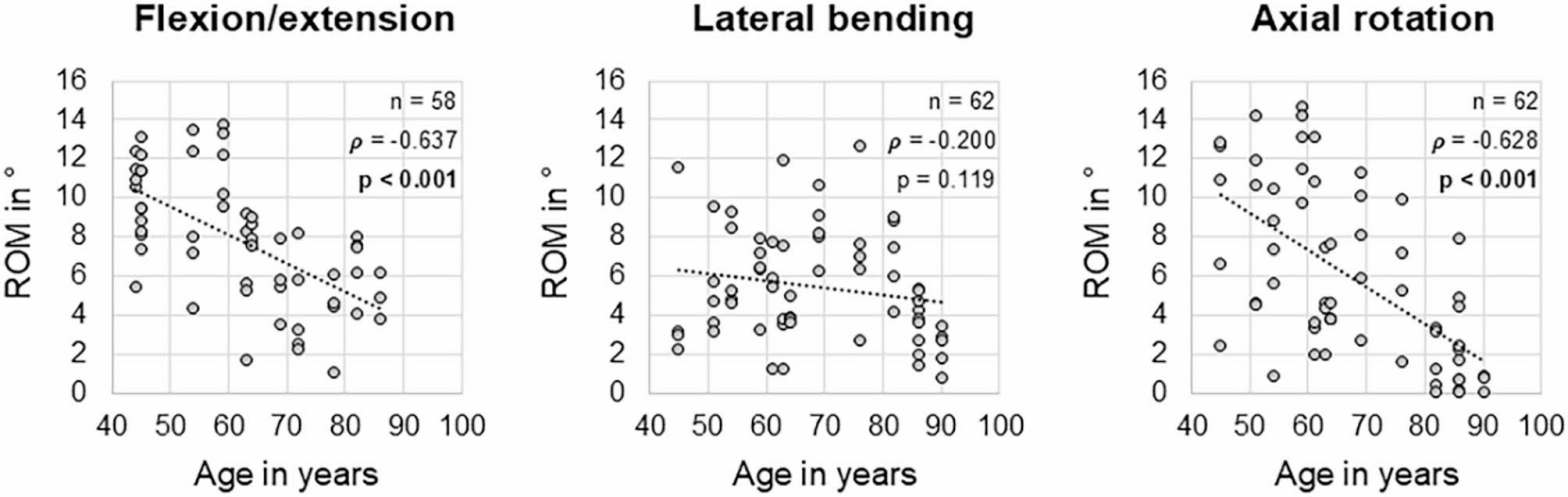
Scatterplot diagrams illustrating the relationship between age and segmental range of motion (ROM) of the cervical spine in the three primary motion planes. Spearman correlation coefficients (*ρ*) and significance values (*p*) are shown in the upper right corner of each diagram.

Furthermore, significant (*p* < 0.001) high negative linear correlations between range of motion and donor age were found in flexion/extension (*ρ* =  − 0.637) and axial rotation (*ρ* =  − 0.628), meaning that the flexibility of the cervical motion segments linearly decreased with increasing donor age (Fig. 6). In lateral bending, the same tendency was detected, while the correlation was low (*ρ* =  − 0.200) and non-significant (*p* = 0.119). The segmental range of motion decreased by 1.5° per decade in flexion/extension and by 1.9° per decade in axial rotation, while it remained almost unchanged in lateral bending (− 0.4° per decade).

### Effects of sex

**Fig. 7:**
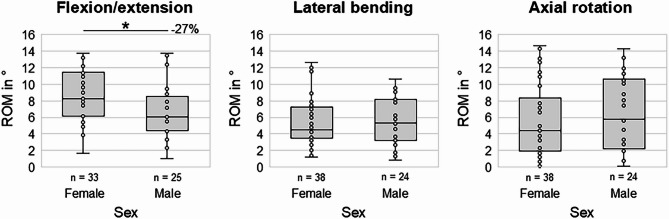
Boxplot diagrams illustrating the effects of sex on the segmental range of motion (ROM) of the cervical spine in the three primary motion planes. *p < 0.05 (Two-sided Mann–Whitney U test).

Donor sex significantly affected the cervical spinal flexibility in flexion/extension (*p* < 0.05), showing a reduced median range of motion in specimens from male donors compared to specimens from female donors by about 27% (Fig. 7). In lateral bending and axial rotation, no significant differences regarding the range of motion were detected between both sexes (*p* > 0.05).

### Effects of segmental level

The ranges of motion were almost comparable between the single motion segments in each of the three primary motion planes, exhibiting no statistical differences between the levels C3-C4 to C6-C7 (*p* > 0.05). The sole statistical differences found were significantly lower range of motion of the C2–C3 level compared to the C3–C4 level in flexion/extension and axial rotation as well as compared to the C4–C5 level in axial rotation (*p* < 0.05) when being evaluated with the two-sided Mann–Whitney U test.

## Discussion

Age-related degenerative changes of the intervertebral disc are highly prevalent in the cervical spine^4,5^ and supposed to contribute to reduced passive cervical spinal mobility and chronic neck pain^6,7^. Findings of previous in vivo studies indicate that the flexibility of the cervical spine is subjected to multiple factors, such as intervertebral disc degeneration, age, segmental level, and potentially also sex. These factors, however, have not been sufficiently elucidated under standardized, reproducible in vitro conditions so far, impeding definite conclusions about their impact on the cervical range of motion and thus limiting the adequate validation of specific experimental and numerical models of the cervical spine. The present in vitro study detected significant effects of intervertebral disc degeneration, age, sex, and segmental level on the range of motion of the cervical spine, which, however, were differently pronounced and strongly dependent on the motion plane. While disc degeneration exhibited significant effects on cervical spinal flexibility in all three primary motion planes, age had no significant effect in lateral bending but showed higher correlation coefficients with the cervical range of motion in flexion/extension and axial rotation compared to disc degeneration. Sex, in contrast, solely affected the cervical range of motion in flexion/extension, whereas the segmental level had only minor influence on cervical spinal flexibility in the present study.

The findings of this in vitro study regarding the negative effects of increasing disc degeneration on the cervical range of motion are consistent with the results of in vivo studies^14–16^. However, in these in vivo studies, solely flexion/extension movements based on lateral radiographs were investigated, while the present in vitro study could additionally show that lateral bending as well as axial rotation are also gradually decreasing with increasing disc degeneration. Moreover, the results of the present study indicate differences regarding the effects of disc degeneration on the individual motion planes: While in flexion/extension, the cervical range of motion was most distinctly reduced from moderate (grade 2) to severe (grade 3) degeneration, the strongest reductions in lateral bending and axial rotation were detected from mildly (grade 1) to moderately (grade 2) degenerated discs. This contrasts with prior findings of our research group on the effects of disc degeneration on the range of motion of the thoracic and lumbar spine, where a strong decrease of the range of motion was already found for mildly degenerated discs of the thoracic spine^26^ and where a gradual decrease in flexion/extension and lateral bending but a gradual increase of the range of motion was detected in axial rotation for the lumbar spine^27^. These differences between the single spinal sections regarding the effects of disc degeneration on resulting flexibility might be attributed to their specific anatomical features, especially regarding disc heights and facet joint morphologies. Particularly, the cervical spine has a relatively strong anterior anulus fibrosus, which determines the segmental range of motion in the sagittal and transverse plane. Degenerative changes, such as disc height loss and anterior osteophyte formation, therefore primarily affect the flexibility in flexion/extension and axial rotation^11,28^, which may explain higher correlation of disc degeneration with the range of motion in these motion planes in the present study. The minor effects of disc degeneration in lateral bending, in contrast, may also be reasoned by the presence of the uncovertebral joints, which were previously shown to significantly contribute to lateral bending flexibility of the cervical spine^29^, potentially attenuating the effects of degenerative disc changes. Another specific feature of cervical discs represents the formation of so-called uncovertebral clefts during the process of disc degeneration, which progress into the core of the disc with advancing age and finally completely split the posterior part of the disc^28,30^, potentially more influencing cervical spinal flexibility in flexion/extension and axial rotation compared to lateral bending.

In order to translate the effects of disc degeneration on cervical spinal flexibility detected in the present in vitro study to the clinics, a clear distinction between clinical and biomechanical instability has to be made. Clinical instability, usually determined from lateral radiographs in full flexion and extension and defined as an increase in combined angular and translational mobility, was found to be increased for medium disc degeneration and decreased for severe disc degeneration in previous clinical trials on the cervical spine^31,32^. These clinical findings confirm the instability hypothesis proposed by Kirkaldy-Willis and Farfan for the lumbar spine comprising a first phase of temporary dysfunction, a second phase of instability, and a third phase of stabilization^33^. However, while the study of Dai and colleagues did not distinguish between angular and translational mobility^32^, Miyazaki and colleagues solely identified an increase in translational mobility for medium disc degeneration, but not for angular mobility, which gradually decreased from medium to severe disc degeneration, being consistent with the findings of the present in vitro study. One explanation for this phenomenon may be that medium disc degeneration, particularly the accompanying disc height loss, entails subluxations of the cervical facet joints, which are stabilized by osteophytes in the later course of disc degeneration^32^, potentially more affecting translational than angular instability. Indeed, in silico studies could show that disc degeneration shifts the instantaneous center of rotation in the cervical spine^34^ and leads to increased loading of the cervical facets joints^35^. As translational movements were not specifically investigated in the present study, these should be additionally evaluated in future in vitro studies on the effects of disc degeneration on cervical flexibility.

Effects of age on the cervical flexibility determined in this in vitro study might be closely related to the degenerative changes occurring during the process of ageing, as higher disc degeneration grades were associated with higher age in the present study (Fig. 2) and as higher prevalence and severity of cervical disc degeneration with increasing age has also been widely reported in literature^4,5,14,31,32,36–39^. Apart from that, several in vivo clinical trials found significant negative influence of age on the range of motion of the cervical spine^15–19^, confirming the results of the present in vitro study. However, while previous in vivo studies determined segmental range of motion decreases of about 1.0° to 1.3° per decade in flexion/extension^15,16^, the present study detected segmental range of motion decreases of 1.5° per decade in flexion/extension, suggesting higher effects of age on the passive flexibility of the cervical spine.

Effects of sex on the cervical range of motion were less pronounced and solely found for flexion/extension in the present study, showing significantly reduced range of motion in specimens from male donors in this motion plane. Indeed, previous in vivo studies reported similar findings regarding a tendency towards higher cervical ranges of motion in females compared to males in flexion/extension^15–19^, while differences were primarily detected for specific segmental levels^15,16^ or for a participant’s age over 70 years^17,19^. While the findings of the present study cannot be explained by other potential influencing factors, as there was no statistically significant effect of sex on disc degeneration and no significant relationship between sex and age or segmental level in this study, large retrospective^5^, prospective^4^, and 10-year longitudinal^37^ studies did also not detect any significant correlations between sex and disc degeneration. However, findings of an in vitro study by Nightingale and colleagues suggest that the male cervical spine is stiffer and stronger than the female in flexion/extension^40^, which might most likely be explained by sex-specific size differences and which might explain the sex differences regarding the flexion/extension range of motion found in the present study.

The segmental level was found to have a minor effect on cervical spinal flexibility in this in vitro study, as solely significantly reduced range of motion of the C2–C3 motion segment was found in flexion/extension and axial rotation when being evaluated by means of pairwise comparisons. While previous in vivo studies investigating cervical segmental ranges of motion did not perform statistical analysis on potential effects of the segmental level, the numbers reported in these studies also suggest a considerably lower range of motion of the C2–C3 motion segment compared to the lower cervical motion segments in flexion/extension^10,15,16^, confirming the findings of the present in vitro study. On the other hand, these results are rather surprising considering that disc degeneration was found to be significantly higher at the lower cervical levels, especially at C5–C6 and C6–C7, compared to the upper cervical levels in the present study (Fig. 2) as well as in previous clinical studies^4,5,31,36–39^, indicating that the level-specific morphological and structural properties of the cervical spine compensate for the effects of disc degeneration on the range of motion.

Due to its in vitro character, the results of the present study are not directly transferable to the in vivo situation. For instance, loading of the specimens by means of pure moments does not directly reflect the physiological loading of the cervical spine by segment-specific muscle forces and additional head weight. However, applying pure moments provides the advantage of clearly defined and thus comparable and reproducible loading conditions, therefore facilitating data interpretation. Moreover, the application of pure moments was shown to allow replication of in vivo cervical spine kinematics^41^. Another limitation of this study is represented by the differences in testing conditions among the three subprojects combined for the present study. However, apart from the use of different measuring devices, which should not have affected the results of the present study due to comparable measuring accuracies, the main differences were regarding the angular deformation rates, which were shown to not influence the range of motion of spinal motion segments^42^. Moreover, plain radiographs were used to determine the grade of intervertebral disc degeneration in the present study, impeding the assessment of facet joint degeneration and potentially limiting the comparability with in vivo studies using magnetic resonance imaging, as gradings of disc degeneration from X-rays and magnetic resonance images were found to differently predict the flexibility of the lumbar spine^43^. However, of the three previous in vivo studies reporting on effects of disc degeneration on cervical flexibility, two also used lateral radiography^15,16^ and solely one magnetic resonance imaging^14^ for degeneration grading. A further limitation of this study is that some groups contained low sample sizes, potentially impeding the detection of further statistical relationships. However, this was primarily the case for grade 3 disc degeneration (n = 3), where still strong statistical differences could be identified and for which the range of motion was generally very low in all three primary motion directions, as severe disc degeneration is usually associated with an immobilization of the motion segment due to very low disc height and bridging osteophytes (Fig. 1)^22^. Thus, it can be expected that an increase in sample sizes would not have significantly changed the results of this study.

## Conclusions

Intervertebral disc degeneration and age show strong effects on the range of motion of the cervical spine, as both are negatively correlated with cervical flexibility and correlated with each other. Sex also affected the cervical range of motion in the present study with the flexion/extension flexibility being higher in females compared to males, while the differences were less pronounced compared to the factors disc degeneration and age. The segmental level exhibited overall low impact on cervical flexibility, but the results of this study and the literature suggest a tendency towards reduced flexibility in the C2–C3 motion segment compared to the lower cervical spinal levels. Overall, the investigated influencing factors appear to be dependent on the motion plane and significantly differ from the thoracic and lumbar spine, underlining the requirement of specific considerations of cervical spinal biomechanics in clinics as well as in future experimental studies. This study provides essential comparative data for the interpretation of past and future in vivo, in vitro, and in silico findings on cervical spinal flexibility as well as for the optimization of biomechanical models of the cervical spine with regard to age, sex, and level-specific intervertebral disc quality.

## Electronic supplementary material

Below is the link to the electronic supplementary material.

Supplementary Material 1

## Data Availability

Data is provided within the manuscript or supplementary information files.
