# Supplementary material for: Intervertebral disc degeneration, age, and sex affect the range of motion of the cervical spine
Source: Sci Rep. 2025 Jul 2;15:23540. doi: 10.1038/s41598-025-07182-4 (PMC12222891; doi:10.1038/s41598-025-07182-4)
Supplement: Supplementary file 1 — Supplementary Material 1 [file 41598_2025_7182_MOESM1_ESM.pdf]

Results

Effects of
intervertebral disc degeneration
on the Range of motion
of the cervical spine

Suppl. Fig. S1

Data set information

19 fresh frozen human specimens (C0-T1)

→ Evaluated levels from C2-C3 to C6-C7

→ Mean age 68±15 years (44-90 years)

11 female, 8 male specimens

* $p < 0.05$ (Pairwise Kruskal-Wallis test with Dunn-Bonferroni post-hoc correction)
\# $p < 0.05$ (Two-sided Mann-Whitney U test)

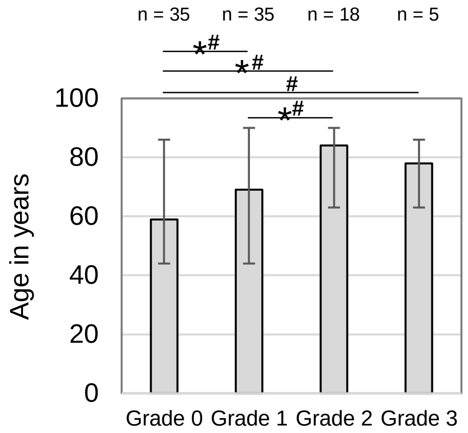

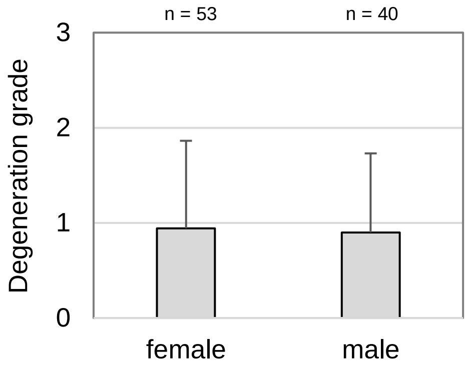

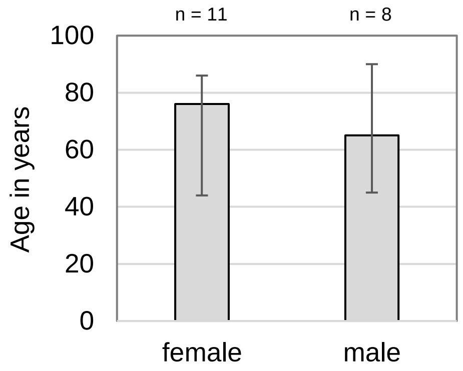

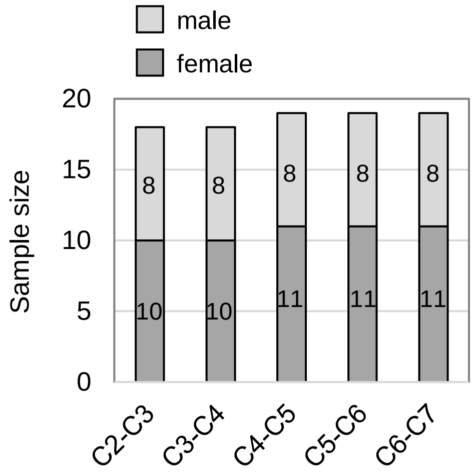

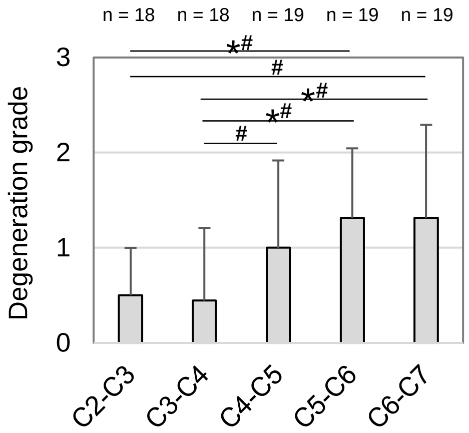

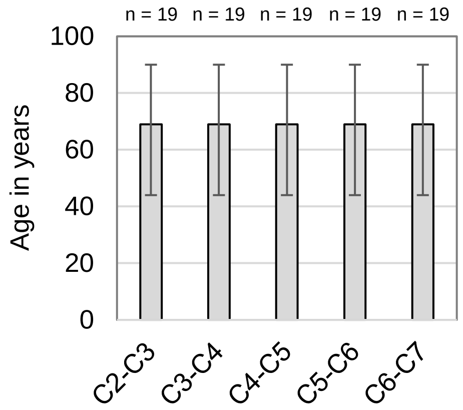

Suppl. Fig. S2

ROM vs. Degeneration grade

Flexion/extension
(segmental levels pooled)

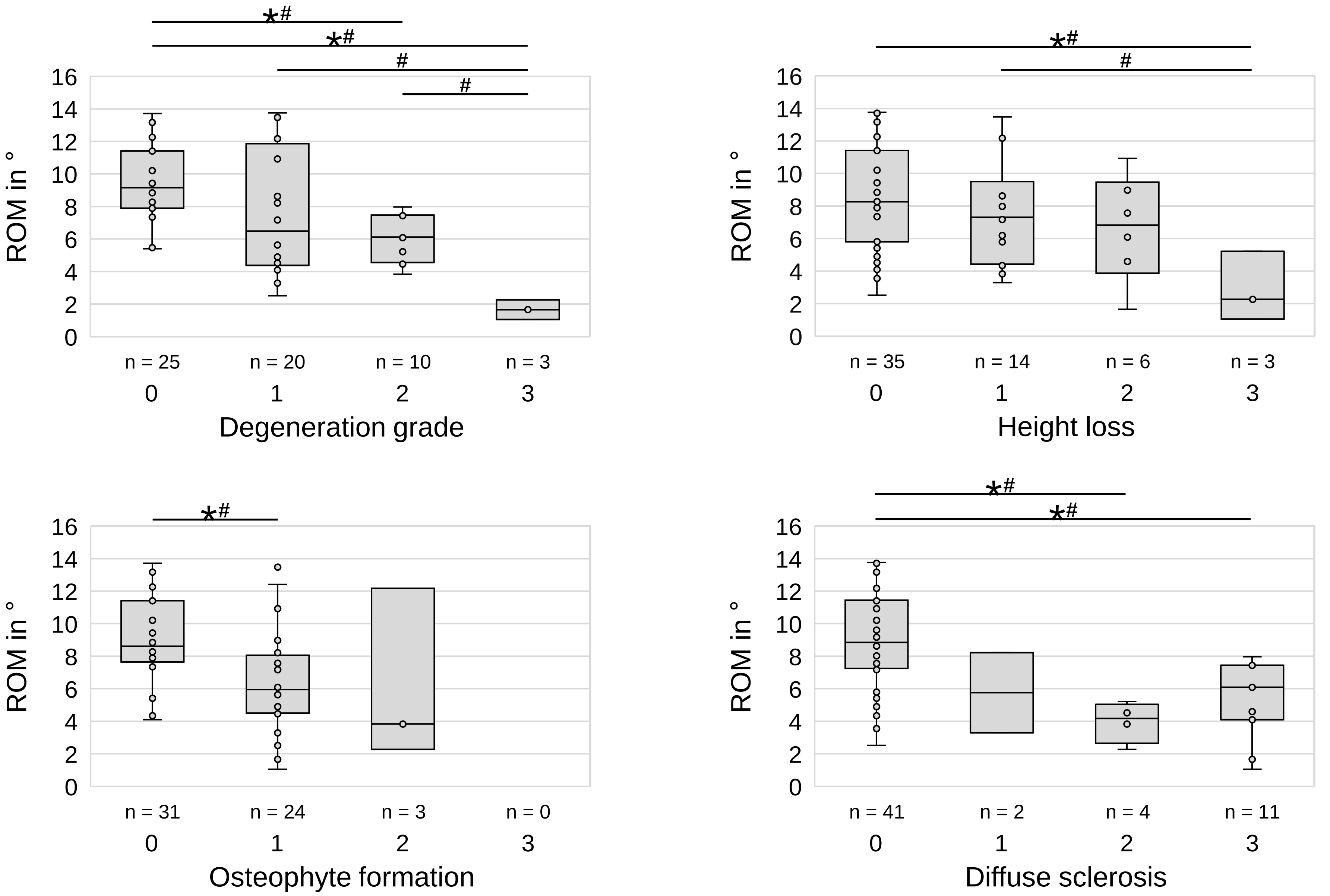

* $p < 0.05$
(Pairwise Kruskal-Wallis test with Dunn-Bonferroni post-hoc correction)

\# $p < 0.05$
(Two-sided Mann-Whitney U test)

Suppl. Fig. S3

ROM vs. Degeneration grade

Lateral bending
(segmental levels pooled)

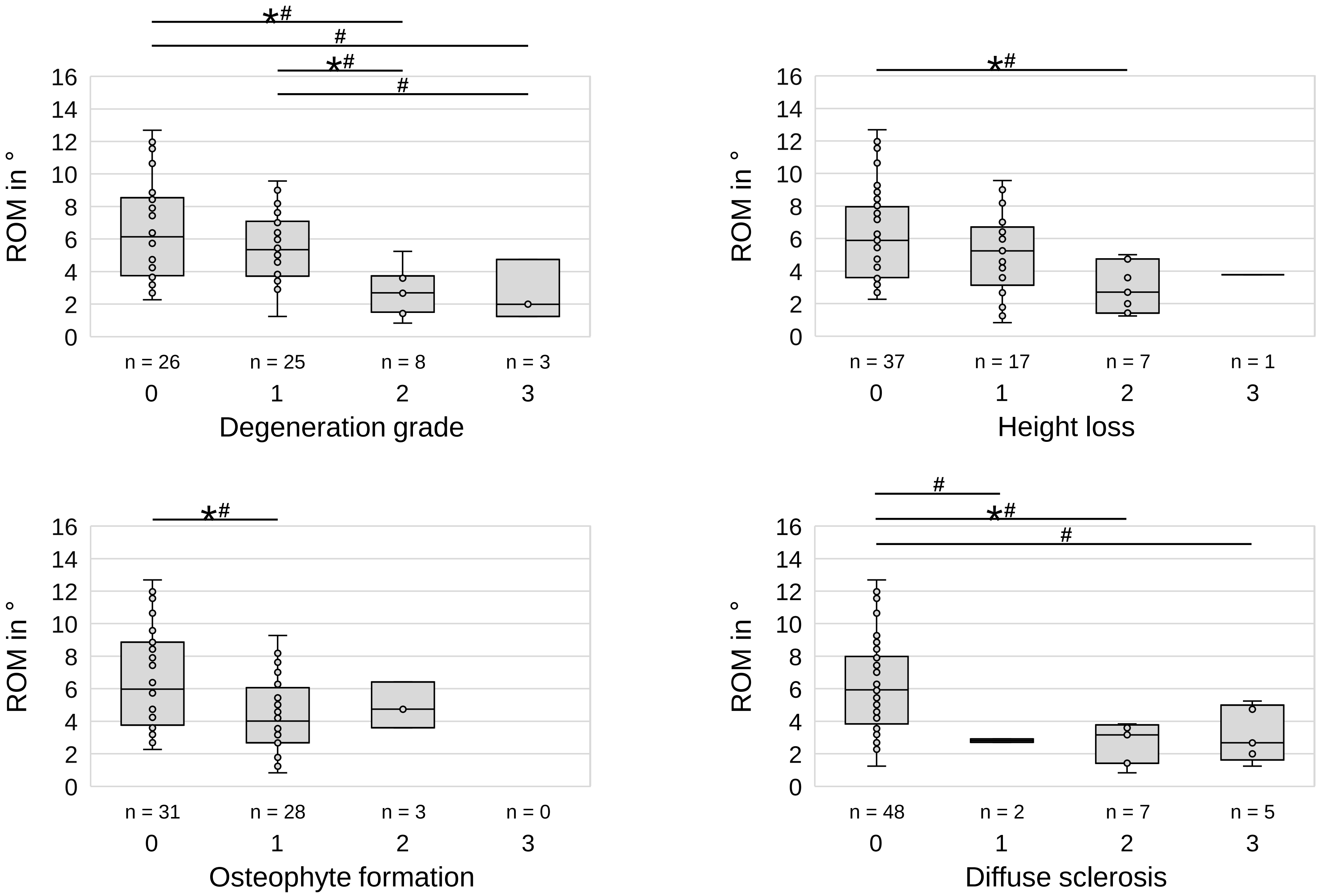

* p < 0.05
(Pairwise Kruskal-Wallis test
with Dunn-Bonferroni
post-hoc correction)

# p < 0.05
(Two-sided
Mann-Whitney U test)

Suppl. Fig. S4

ROM vs. Degeneration grade

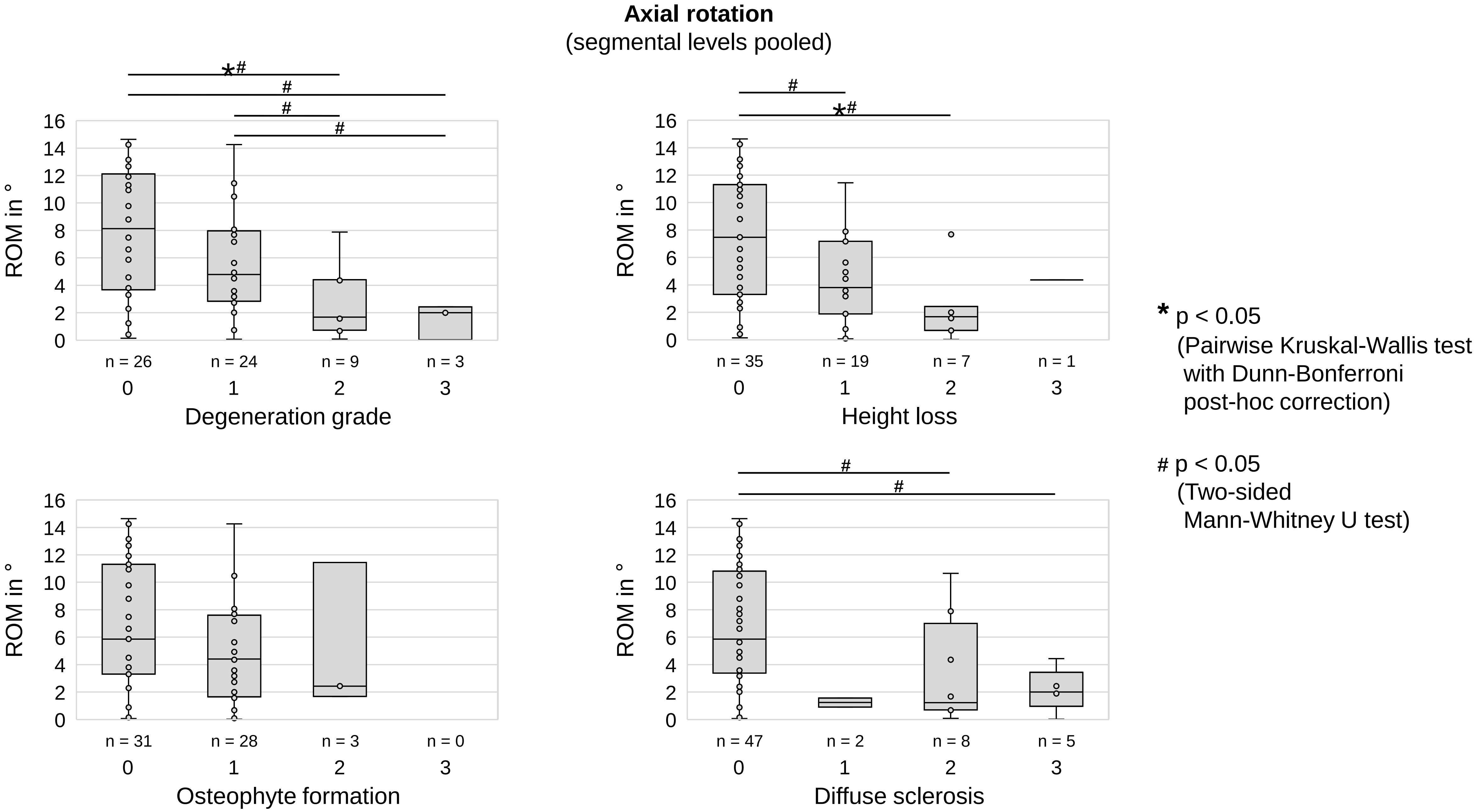

Suppl. Fig. S5

ROM vs. Degeneration grade

Flexion/extension
(all data points)

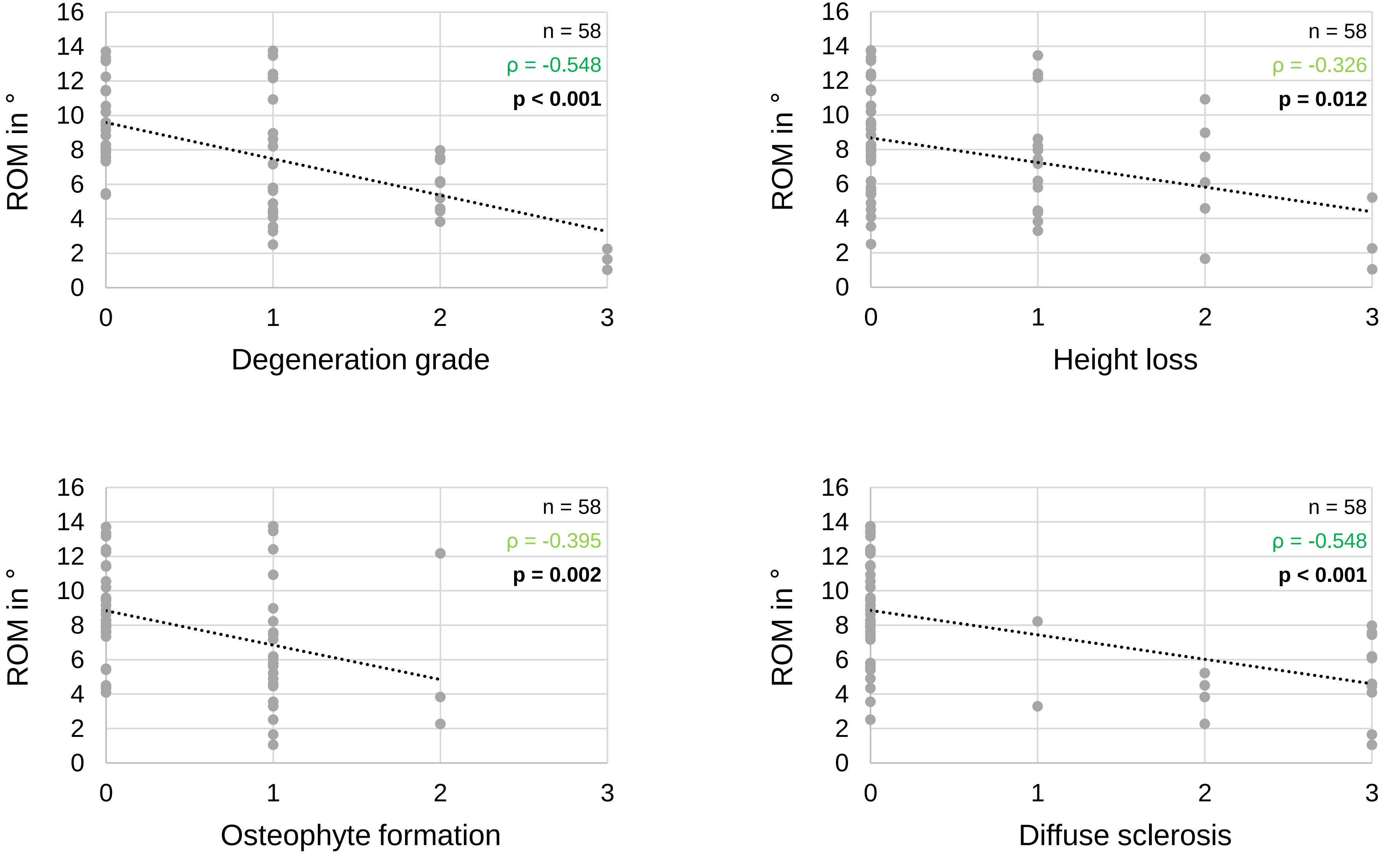

ρ Spearman correlation coefficient

Cohen 1988:

$|\rho| \leq 0.1$	no linear correlation
$0.1 < |\rho| \leq 0.3$	low linear correlation
$0.3 < |r| \leq 0.5$	medium linear correlation
$|r| > 0.5$	high linear correlation

Suppl. Fig. S6

ROM vs. Degeneration grade

Lateral bending
(all data points)

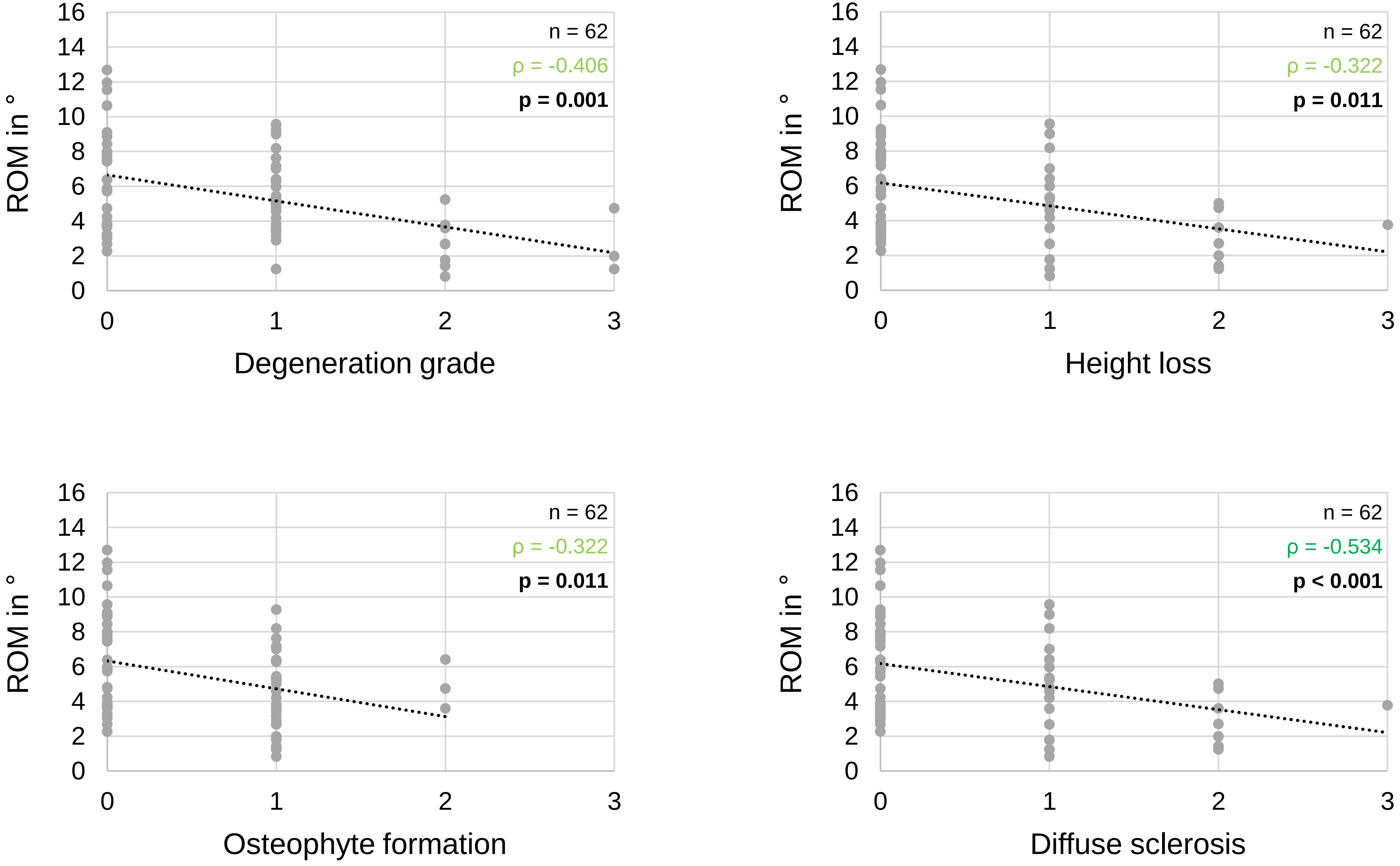

ρ Spearman correlation coefficient

Cohen 1988:

|ρ| ≤ 0.1	no linear correlation
0.1 < |ρ| ≤ 0.3	low linear correlation
0.3 < |r| ≤ 0.5	medium linear correlation
|r| > 0.5	high linear correlation

Suppl. Fig. S7

ROM vs. Degeneration grade

Axial rotation
(all data points)

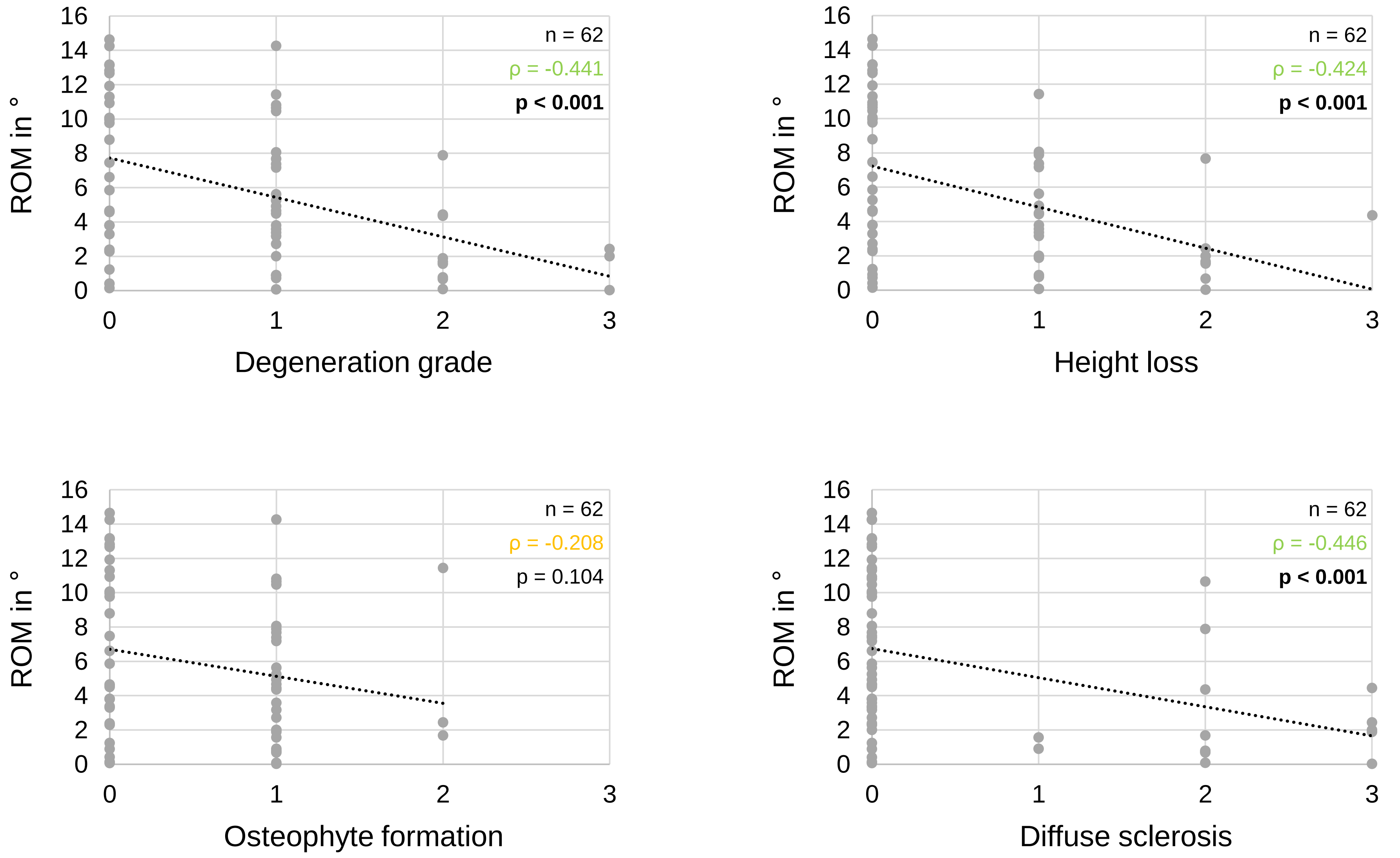

ρ Spearman correlation coefficient

Cohen 1988:

|ρ| ≤ 0.1	no linear correlation
0.1 < |ρ| ≤ 0.3	low linear correlation
0.3 < |r| ≤ 0.5	medium linear correlation
|r| > 0.5	high linear correlation

Suppl. Fig. S8

ROM vs. Age
(all data points)

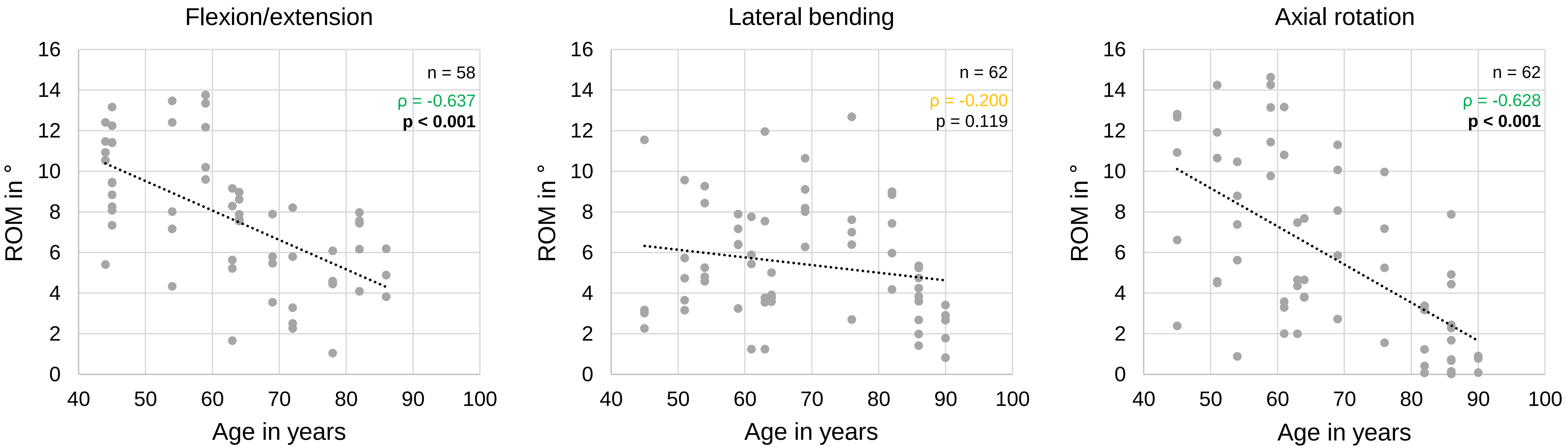

ρ Spearman correlation coefficient

Cohen 1988:

$\|\rho\| \leq 0.1$	no linear correlation
$0.1 < \|\rho\| \leq 0.3$	low linear correlation
$0.3 < \|r\| \leq 0.5$	medium linear correlation
$\|r\| > 0.5$	high linear correlation

Suppl. Fig. S9

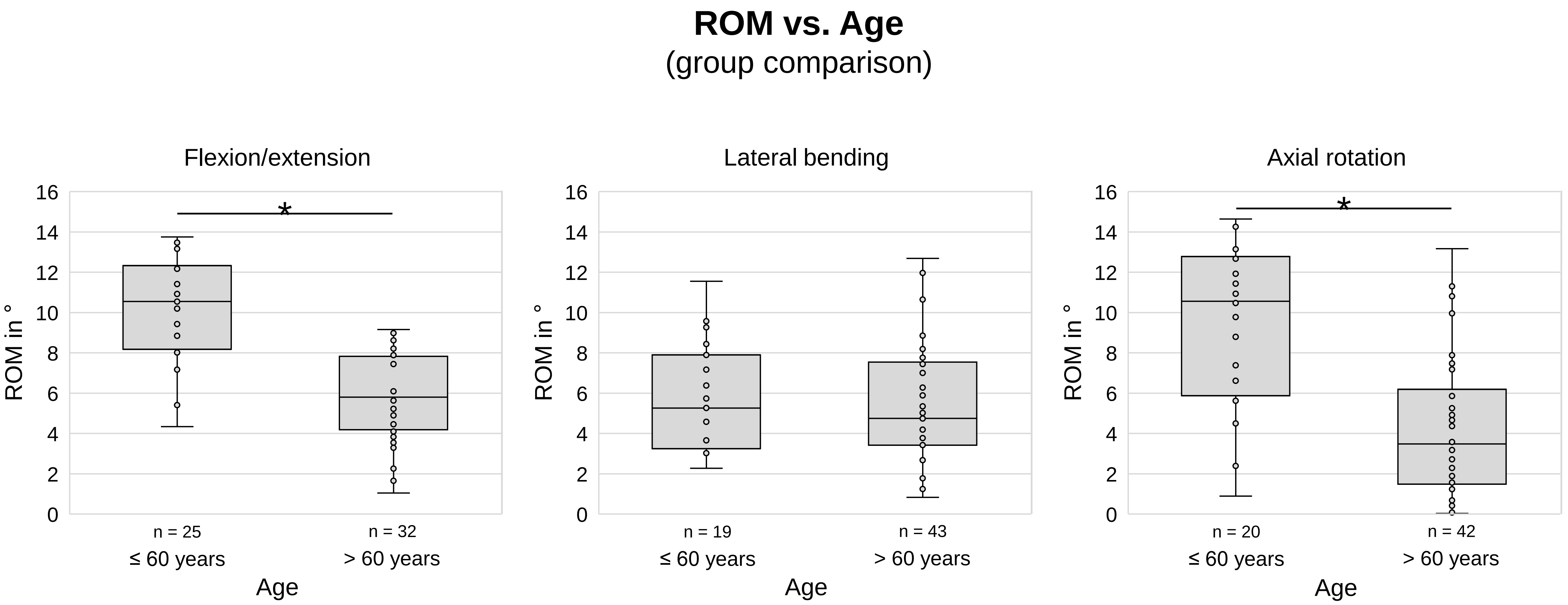

***** $p < 0.05$ (Two-sided Mann-Whitney U test)

Suppl. Fig. S10

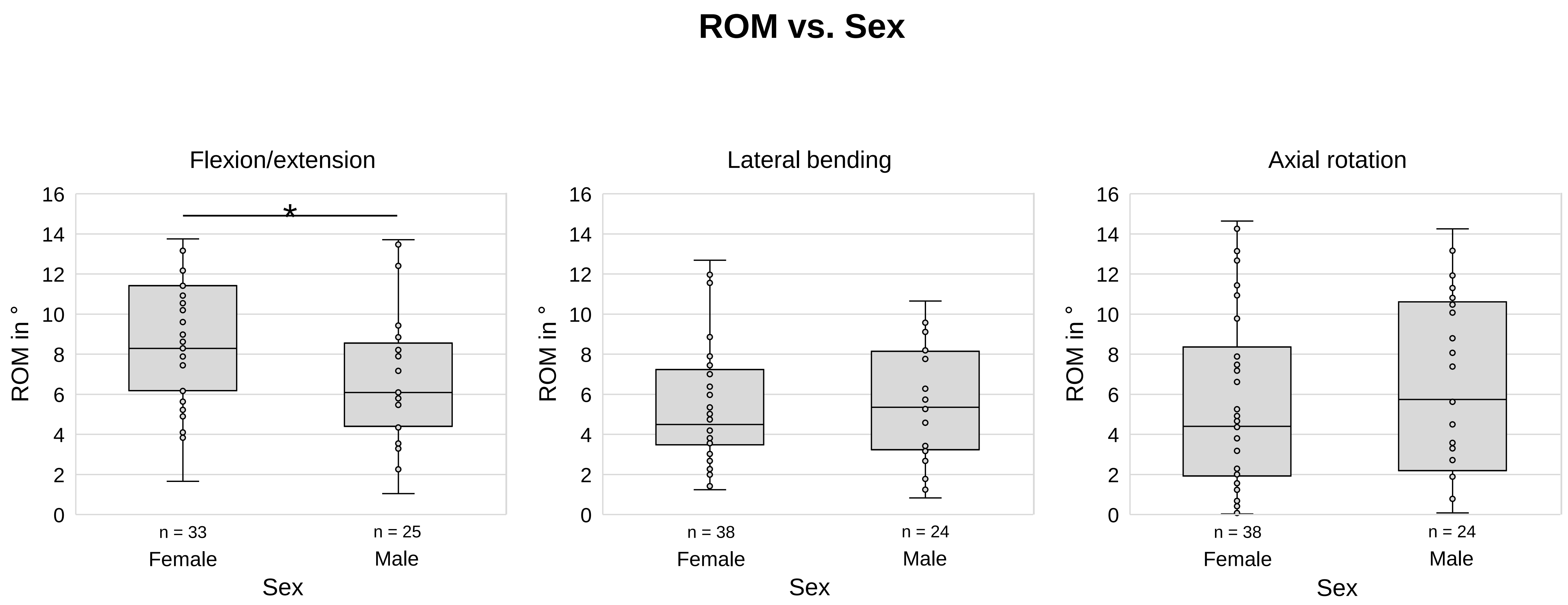

***** $p < 0.05$ (Two-sided Mann-Whitney U test)

Suppl. Fig. S11

ROM vs. Segmental level

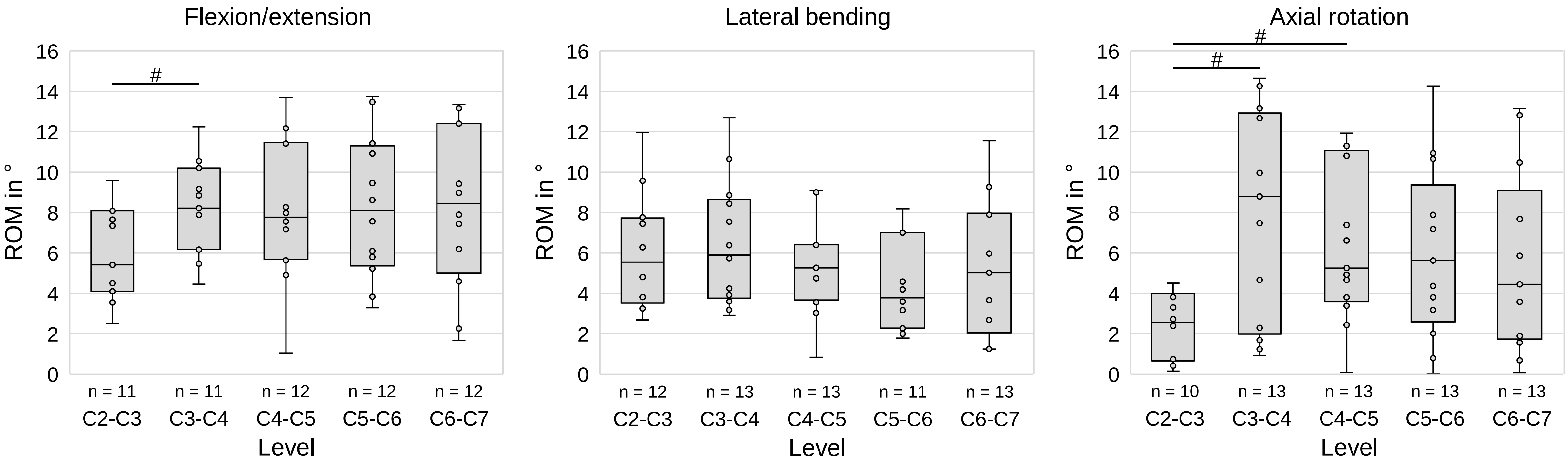

* $p < 0.05$ (Pairwise Kruskal-Wallis test with Dunn-Bonferroni post-hoc correction)

\# $p < 0.05$ (Two-sided Mann-Whitney U test)
